# Cerebrospinal Fluid Proteomic Profiling Reveals Proteins Associated with Neuroinflammatory Response in COVID-19 Patients

**DOI:** 10.1021/acsomega.5c00707

**Published:** 2025-06-11

**Authors:** Juliana Ramos de Andrade, Josivan Barbosa de Farias, Maria Luiza de Lima Vitorino, Fernando Tenório Travassos, Roberto Afonso da Silva, José Luiz de Lima Filho, Marcelo Moraes Valença

**Affiliations:** 1 Keizo Asami Institute, 28116 Federal University of Pernambuco. Av. Prof. Moraes Rego, Recife ,Pernambuco 50670-901, Brazil; 2 Fernando Travassos Laboratory, Empresarial Thomas Edison - Av. Gov. Agamenon Magalhães, 4775 - Boa Vista, Recife, Pernambuco 50070-160, Brazil

## Abstract

The COVID-19 pandemic has highlighted the diverse clinical manifestations of SARS-CoV-2 infection, including neurological complications. This study investigates cerebrospinal fluid (CSF) proteomic profiles to identify proteins associated with neuroinflammatory processes in COVID-19. CSF samples from 11 critically ill patients and 5 COVID-19-negative controls were analyzed using high-resolution liquid chromatography-tandem mass spectrometry. A total of 203 proteins were identified, of which 76 exhibited differential expression peptides (DEPs). Proteins involved in coagulation (fibrinogen alpha and beta chains, prothrombin) and immune responses (complement cascade components, immunoglobulin heavy constant gamma, kappa, and lambda subunits) were significantly upregulated in COVID-19 patients. In contrast, proteins involved in antioxidant defense (e.g., superoxide dismutase) and neural maintenance (e.g., neural cell adhesion molecule 1, Neuronal cell adhesion molecule) were significantly downregulated. Functional annotation revealed enriched pathways associated with hemostasis, immune regulation, and neuroinflammatory responses. Protein–protein interaction analysis highlighted interactions among complement and coagulation cascade components, underscoring their roles in inflammation and potential thrombotic complications. These findings suggest that SARS-CoV-2 infection induces significant alterations in the CSF proteome, reflecting neuroinflammatory and oxidative stress mechanisms. Identifying these proteins and their association with diverse pathophysiological mechanisms provides insights into the neurological impact of COVID-19 and may serve as therapeutic targets and potential biomarkers. Further studies are needed to validate these findings and explore their clinical implications in post-COVID-19 neurological syndromes.

## Graphical abstract

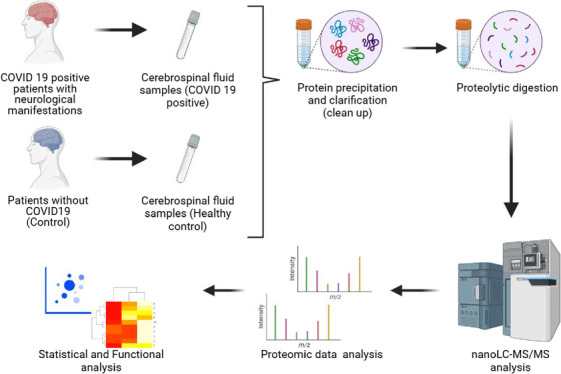

## Introduction

The emergence of novel coronavirus SARS-CoV-2, which causes the disease COVID-19, has led to a global health crisis with a broad spectrum of clinical manifestations. Among these, neurological symptoms have been reported in a significant proportion of patients, ranging from mild headaches to severe complications such as encephalitis and acute disseminated encephalomyelitis (ADEM).^1^ The diversity in the pathogenesis of these neurological manifestations generates the need for new molecular insights that can inform diagnosis, prognosis, and better therapeutic strategies.^2^

Proteomics, the large-scale study of proteomes, all proteins expressed by a cell, tissue, or organism, provides a powerful approach to understanding the mechanisms of numerous diseases.^3^ Cerebrospinal fluid (CSF) is a particularly informative biofluid for studying CNS pathologies because it is in direct contact with the extracellular space of the brain and spinal cord, mainly reflecting biochemical changes in the CNS.^2^ CSF proteomic analysis in the context of COVID-19 offers a unique opportunity to discover the molecular bases associated with the impact of the virus on the CNS according to proteins of important pathophysiological pathways.^4,5^

This article presents a comparative proteomic analysis of CSF from patients with acute COVID-19 with neurological involvement versus a healthy control group. By utilizing high-throughput proteomic techniques, we aim to identify differential protein expression patterns and pathway alterations that could shed light on the neuropathophysiological processes in COVID-19. Furthermore, identifying unique protein signatures in the CSF of COVID-19 patients could reveal biomarkers of neurological involvement and facilitate a better understanding of the disease’s heterogeneity.

Given the novelty of COVID-19, there is a limited but rapidly growing body of literature on CNS involvement. Preliminary studies have suggested that the virus can induce a host immune response that may lead to neuroinflammation, potentially contributing to neurological symptoms.^3^ However, comprehensive proteomic profiles of CSF from COVID-19 patients with neurological symptoms have yet to be extensively characterized. This study intends to fill this gap by providing a detailed proteomic landscape and identifying potential targets for further investigation.

## Results

A total of 203 proteins were identified, where according to the abundance values and statistical analyses performed (FDR: 0.01 and *p*-value ajusted: <0.05), 76 were found as differentially expressed—DEPs. Of these DEPs, 45 were considered upregulated, and 31 were considered downregulated (Figure 1). It was not possible to identify any protein exclusive to the samples studied. Of the positively regulated proteins, some are involved in the coagulation cascade, such as haptoglobin (HP), prothrombin (F2), fibrinogen alpha chain (FGA), and fibrinogen beta chain (FGB). The post hoc effect size analysis revealed large to substantial effects: 2.7 for haptoglobin, 3.4 for prothrombin, 6.2 for fibrinogen alpha chain, and 9.4 for fibrinogen beta chain.

**Figure 1:**
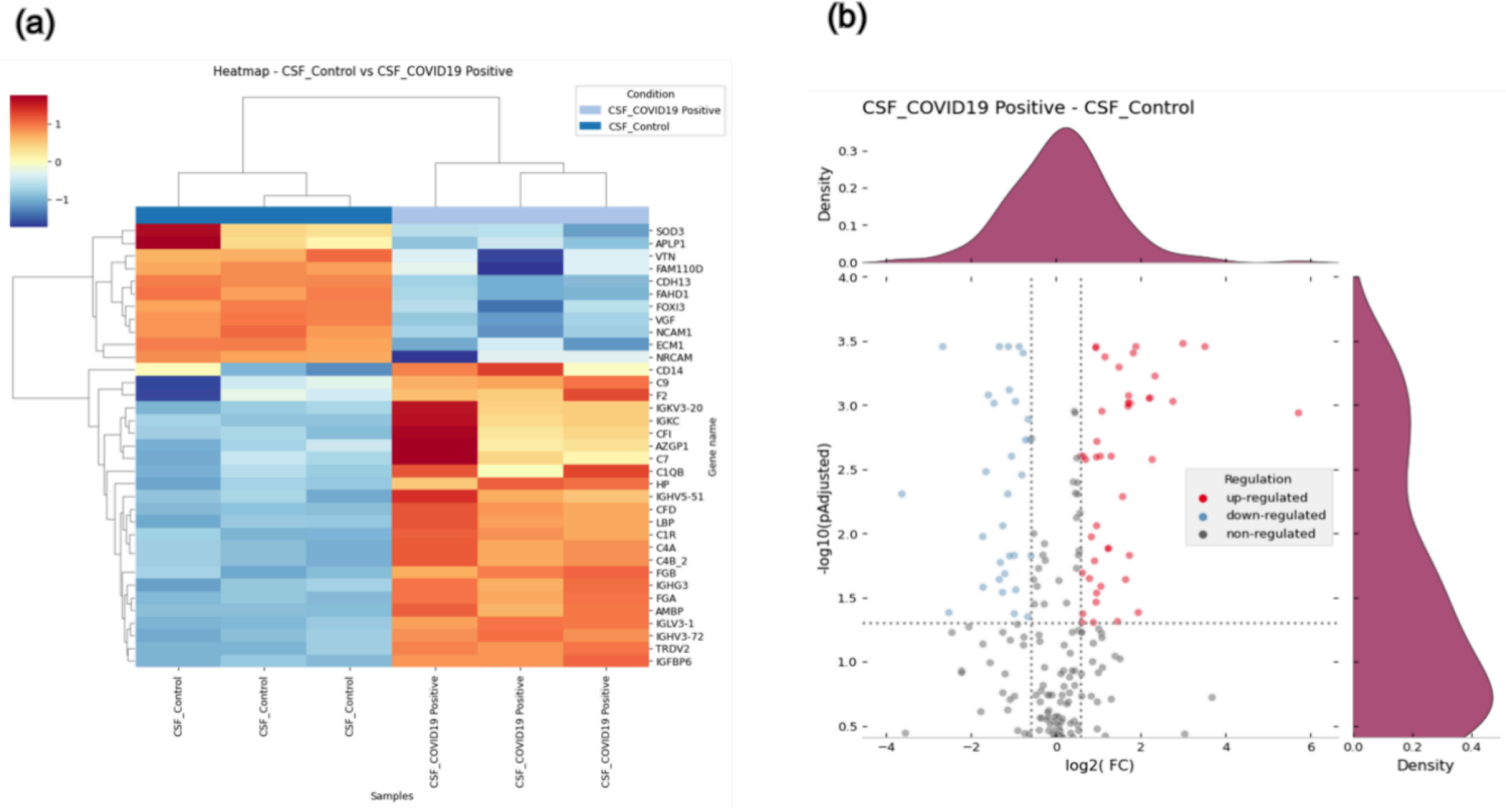
Distribution of differentially expressed proteins (DEPs) in clinical CSF and COVID-19 samples. (a) Heatmap showing the DEP’s (intensity on the color scale according to the *Z* score values in each replicate of the test and control samples). (b) Volcano plot of all DEPs according to their significance values (on the *x*-axis, we used: log2 of folder charge and on the *y*-axis: −log10 *p*-value). CSF_COVID-19 (positive) replicates: CSF_P_01, CSF_P_02 and CSF_P_03. CSF_Control replicates (negative): CSF_N_01, CSF_N_02, and CSF_N_03.

Others involved in immunological processes have also been shown to be upregulated, such as the T-cell receptor delta variable 2 (TRDV2), immunoglobulin heavy constant gamma (IGHG3), immunoglobulin kappa constant (IGKC), immunoglobulin lambda variable 3–1 (IGLV3–1), immunoglobulin kappa variable 3–20 (IGKV3–20), and immunoglobulin heavy variable 5–51 (IGHV5–51), and the complement cascade: complement C1r (C1R), complement factor D (CFD), complement C4-A (C4A), complement C4–B (C4B), complement factor I (CFI), component of complement C7 (C7), complement C9 (C9), and subunit B of the C1q subcomponent of complement (C1QB). Other proteins associated with neuropathological processes were also identified with increased abundance, such as lipopolysaccharide-binding protein (LBP) and zinc alpha-2 glycoprotein (AZGP1).

While those with negative regulation were proteins related to the antioxidant system, such as extracellular superoxide dismutase [Cu–Zn] (SOD3) and those related to the maturation and maintenance of neurons: neural cell adhesion molecule 1 (NCAM1), neuronal cells (NRCAM), and amyloid beta precursor like protein 1 (APLP1). In Table S1, all differentially expressed proteins are listed.

In the correlation analyses, we evaluated the values between the sample replicates of each group. We observed a positive correlation between the replicates of each sample group. The Pearson correlation values between the test replicates (CSF_COVID-19 Positive) presented values of ≤1.0, with a variation between 0.86 and 0.98. In the control replicates (CSF_Control/COVID-19 negative), the values were between 0.98 and 1.0. When crossing the replicates of different groups, the values fall to a range of up to 0.75 (Figure 2a). Thus, demonstrating that there is a good correlation between the replicates of each sample group.

**Figure 2:**
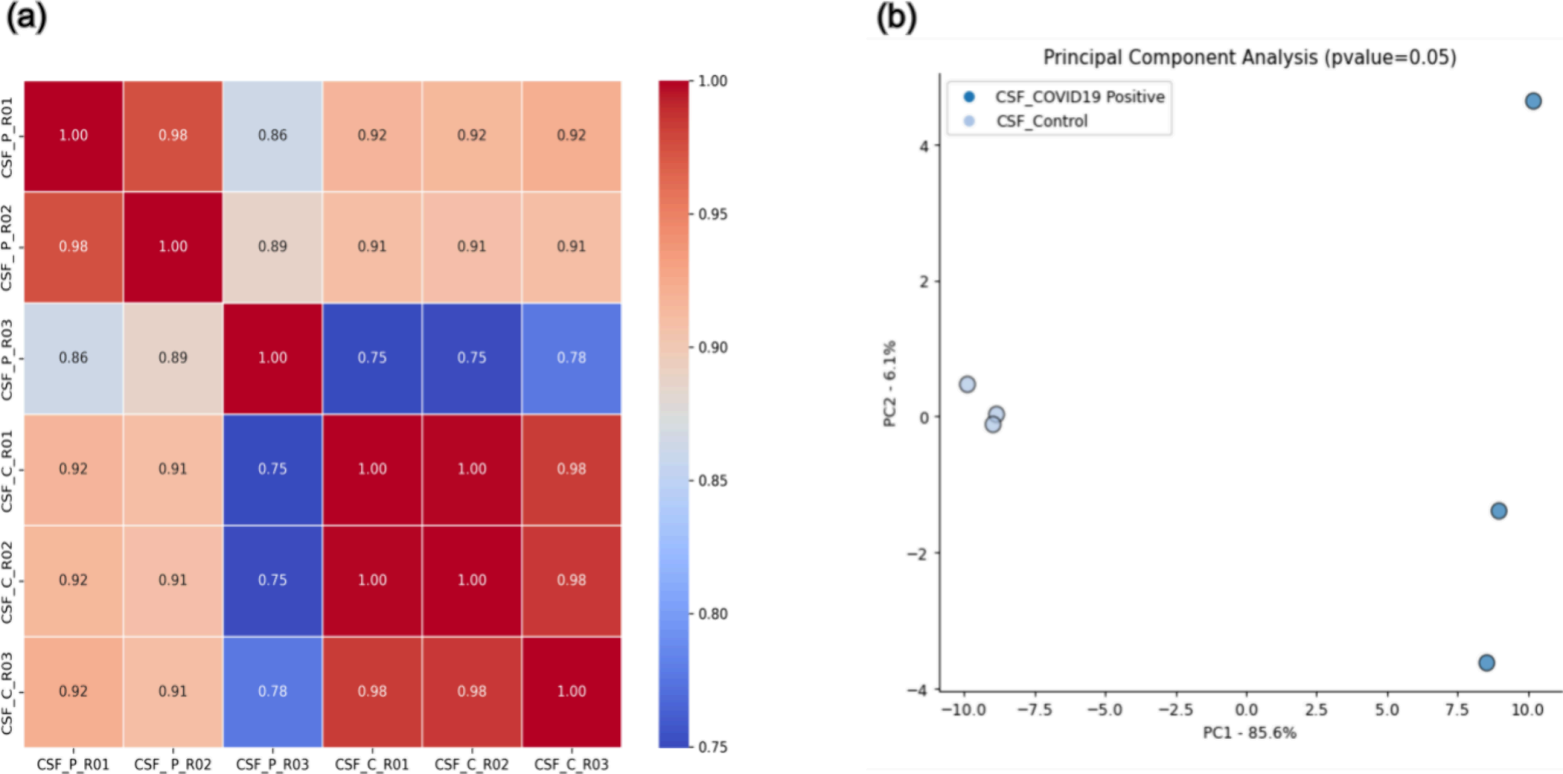
(a) MultiScatter plot with Pearson’s Correlation values between replicates of Test (CSF_COVID-19 positive) and CSF_Control (COVID-19 negative) samples. Correlation values on scales of 0.75 and 1.00. CSF_COVID-19 (positive) replicates: CSF_P_R01, CSF_P_R02, and CSF_P_R03. CSF_Control replicates (negative): CSF_N_R01, CSF_N_R02 and CSF_N_R03. (b) Principal component analysis (PCA).

In the principal component analysis (PCA), we noticed good separation between the sample groups. Furthermore, there was no overlap of replicates between the CSF Positive group and the control, and the percentage obtained was above 90%, demonstrating a good separation profile between the sample replicates (Figure 2b).

Our functional annotation analyses by gene ontology revealed biological processes such as hemostasis, negative regulation of coagulation, and fibrinolysis (Figure 3a). Some of the identified proteins are associated with cellular components, such as cellular secretory products and vesicle transport (Figure 3b). And activities directly linked to proteases and antigenic groups were defined as the main molecular functions (Figure 3c).

**Figure 3:**
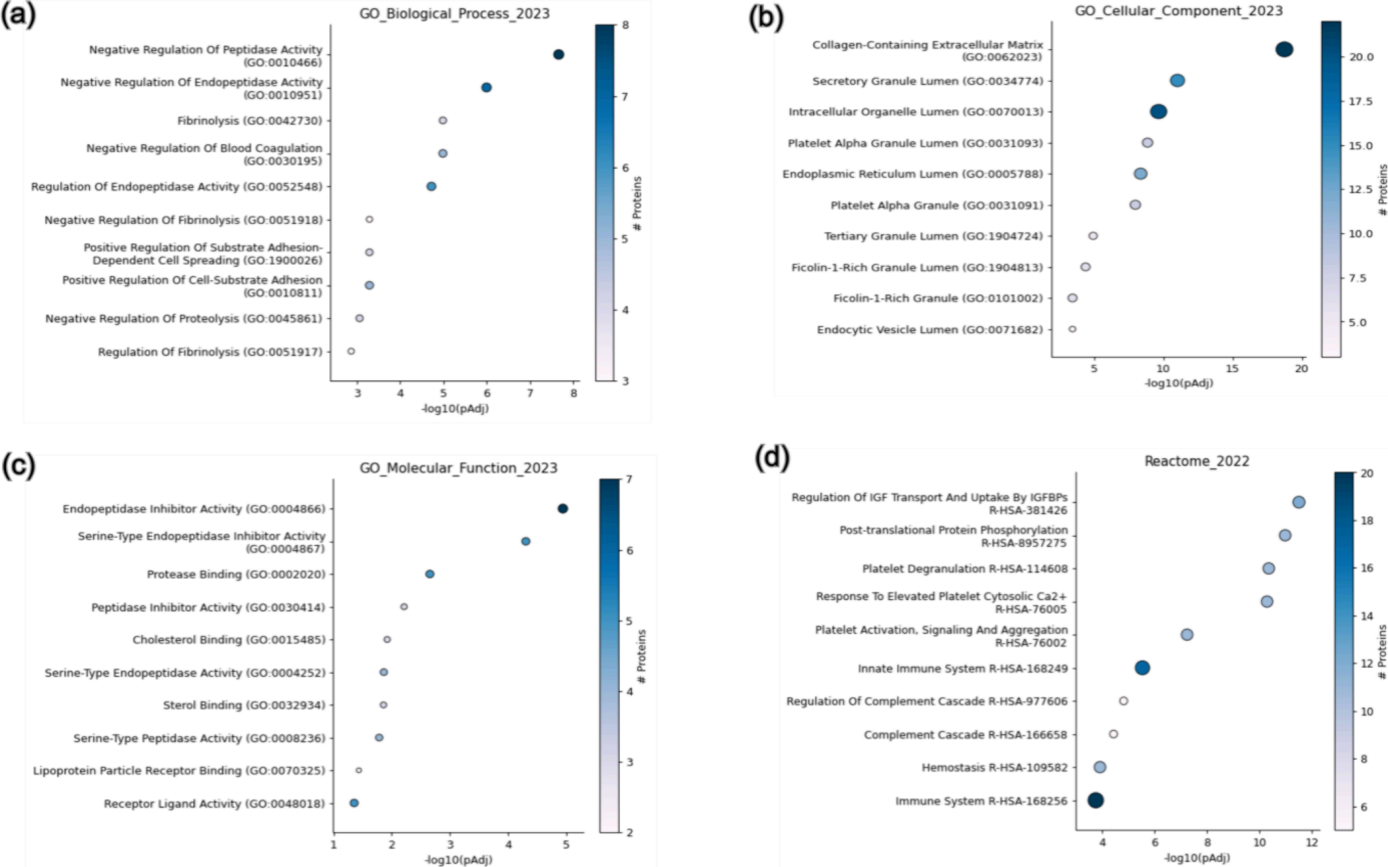
Functional annotation of DEPs. (a) Biological processes are characterized by Gene Ontology (GO). (b) Cellular components are characterized by Gene Ontology. (c) Molecular function by Gene Ontology. (d) REACTOME pathways.

Biological pathways based on the REACTOME database were identified, many associated with processes such as regulation of the complement cascade, coagulation cascade (fibrin clot formation, activation, signaling and platelet aggregation), and hemostasis, in addition to others associated with immunological processes, such as in the regulation of the innate immune system (Figure 3d).

In protein–protein interactions, many of those associated with the complement cascade, such as CFD, C1QB, C9, C4A, and C4B, demonstrate strong interaction, which can be observed in altered regulation events in the classical and alternative complement pathways, in addition to proteins associated with neural maintenance (NCAM1, NRCAM), as well as those involved in hemostatic processes (Figure 4a).

**Figure 4:**
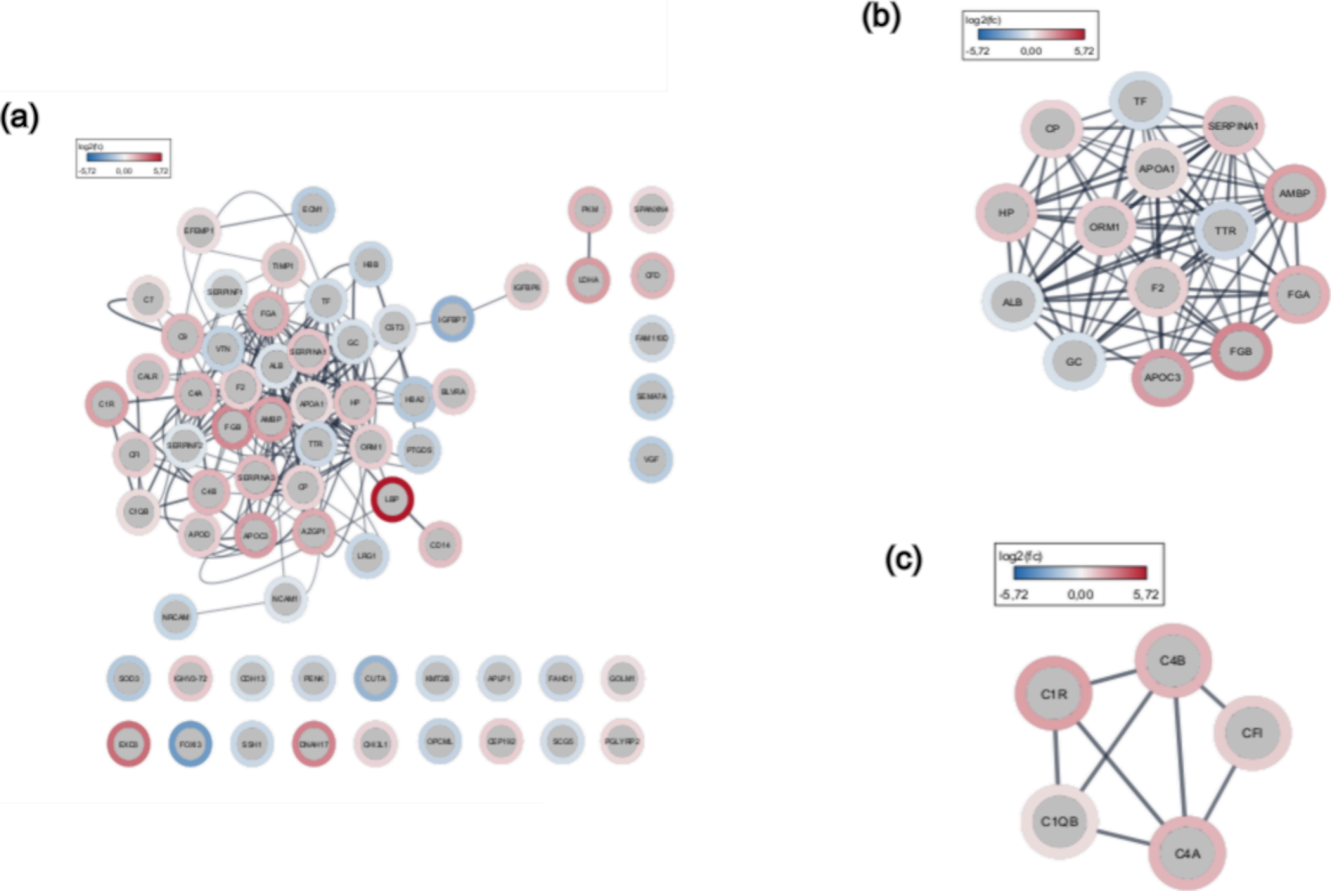
Protein–protein interactions (PPI) were measured by Cytoscape. (a) Interaction network between differentially expressed proteins (Omics visualizer plugin via STRING database). (b) Cluster 1 of proteins with the highest interaction in the core PPI network (via MCODE plugin). (C) Cluster 2 of proteins with the highest interaction in the core PPI network (via MCODE plugin). Upregulated proteins represented in red and downregulated proteins in blue. Confidence score: 0.70.

Furthermore, insulin-like growth factor binding protein 7 (IGFBP7), which plays an important role in regulating neuroinflammatory processes in response to brain injury and was downregulated, showed direct interaction with insulin-like growth factor binding protein 5 (IGFBP5), which, in turn, was upregulated.

Proteins involved in coagulation processes, such as fibrinogen alpha and beta chains (FGA and FGB), whose increase is often associated with acute inflammatory states, have been shown to interact with other coagulation proteins, such as alpha-2 antiplasmin (SERPINF2) and prothrombin (F2), generating a cluster of proteins that demonstrate strong interaction via MCODE analysis with a confidence score: 0.70 (Figure 4b).

Another protein cluster also showed greater interaction (cluster 2: confidence score: 0.70), characterized by the complementary proteins, C1QB, C1R, C4A, C4B, and CFI, a detail that was observed as a characteristic factor of interaction in the main PPI network (Figure 4c).

## Discussion

The CSF proteomic analysis of COVID-19 patients revealed significant alterations in protein expression, providing insights into the neuroinflammatory and coagulation disturbances associated with the disease. The identification of 76 differentially expressed proteins (DEPs), with distinct patterns of up- and down-regulation, suggests that SARS-CoV-2 infection induces complex molecular changes in the CNS.

Among the upregulated proteins, several components of the complement cascade, including complement C1r (C1R), complement factor D (CFD), complement C4-A (C4A), complement C4–B (C4B), complement factor I (CFI), and complement C9 (C9), were significantly increased. The overactivation of the complement system has been implicated in neuroinflammation and blood-brain barrier (BBB) dysfunction, as previously reported by Reinhold et al.,^5^ who identified complement activation in COVID-19 CSF samples as a potential contributor to neuronal damage and glial activation. Similar findings were reported by Domingues et al.,^4^ suggesting that increased complement activity could exacerbate neuroinflammatory responses in severe cases of COVID-19. Additionally, our study identified an upregulation of the T-cell receptor delta variable 2, which plays a role in antigen recognition, reinforcing the presence of a sustained immune response within the CNS, consistent with observations from Attaf et al.^6^

The elevated levels of immunoglobulin chains detected in the CSF of COVID-19 patients further support the hypothesis of BBB dysfunction. Selective transport mechanisms typically regulate CSF immunoglobulin concentrations, and an increase in their abundance suggests either intrathecal synthesis or increased BBB permeability, allowing for passive diffusion from systemic circulation. This phenomenon has been observed in other neuroinflammatory conditions and was noted by Wang et al.,^7^ who reported elevated immunoglobulin levels in COVID-19 CSF samples. Significantly, Wang et al.^7^ detected SARS-CoV-2 RNA in the CSF using NGS, whereas we did not perform viral RNA detection in our CSF samples. The presence of SARS-CoV-2 RNA in CSF has been reported, but it remains rare and inconsistently documented in the literature. While a few case studies have confirmed its detection in patients with severe neurological symptoms or long COVID,^8,9^ more extensive studies indicate that direct viral invasion of the CNS is uncommon. Most CSF analyses, including those by Boesl et al.^10^ reveal normal or only mildly altered parameters, suggesting that neurological manifestations are more likely driven by systemic immune dysregulation, inflammatory mediators, and blood-CSF barrier dysfunction rather than direct viral infection. Therefore, although we acknowledge the absence of RNA testing as a limitation, our findings remain consistent with current evidence indicating that proteomic and immunological alterations in the CSF can occur independently of detectable viral RNA.

The presence of immunoglobulins, along with an activated complement system, suggests that SARS-CoV-2 infection may induce an immune-mediated response within the CNS, potentially driven by peripheral immune activation rather than direct viral invasion, in line with the findings of Reinhold et al.^5^

Moreover, the subclass distribution of immunoglobulins in the CSF may offer a deeper perspective into the nature of the neuroinflammatory response.^11–13^ Elevated levels of IgG1 and IgG3, in particular, have been associated with pro-inflammatory activity and a stronger capacity to activate the complement cascade, potentially exacerbating CNS inflammation.^12,13^ Additionally, studies have indicated that BBB disruption in COVID-19 patients may be mediated by cytokine-induced endothelial dysfunction, facilitating the passage of immunoglobulins and other plasma proteins into the CSF.^14^ This compromised barrier integrity not only permits the infiltration of immune mediators but may also contribute to neuronal injury and altered neurological function, reinforcing the role of systemic immune dysregulation in CNS pathophysiology during SARS-CoV-2 infection.^15,16^

Another highly upregulated protein in our study was the Lipopolysaccharide-Binding Protein (LBP), which plays a crucial role in innate immunity by facilitating the recognition of lipopolysaccharides (LPS) from bacterial endotoxins. LBP amplifies macrophage activation and enhances the production of pro-inflammatory cytokines. Elevated LBP levels in CSF suggest an exacerbated inflammatory state in patients with COVID-19. This aligns with the findings of Fang et al.,^17^ who reported that LBP can inhibit monoamine biosynthesis, potentially disrupting essential neuromodulatory pathways. LBP has been shown to act as an endogenous inhibitor of dopamine β-hydroxylase (DBH) and aromatic l-amino acid decarboxylase (DDC), two key enzymes involved in dopamine and serotonin synthesis.^7,17^ Given that dopaminergic dysfunction has been implicated in neuropsychiatric symptoms, such as fatigue and cognitive impairment, the significant upregulation of LBP observed in our findings may contribute to post-COVID neurological sequelae. Additionally, LBP has been associated with increased neuroinflammation in neurodegenerative diseases, further supporting its role in exacerbating long-term CNS dysfunction.^7^

Proteins associated with the coagulation cascade, such as fibrinogen alpha chain (FGA), fibrinogen beta chain (FGB), prothrombin (F2), and haptoglobin (HP), were significantly upregulated in the CSF of COVID-19 patients, suggesting a hypercoagulable state that could contribute to microvascular thrombosis within the CNS. Similar findings were reported by Maity et al.,^19^ who identified increased coagulation markers in COVID-19 CSF samples, highlighting the interplay between coagulation and neuroinflammation. Hypercoagulability in COVID-19 has been linked to endothelial dysfunction induced by cytokine storms, which may facilitate fibrin deposition in the brain. As observed in neurodegenerative diseases such as Alzheimer’s, where fibrin interacts with amyloid-β (Aβ) receptors to form resistant fibrin clots, fibrin accumulation in COVID-19 patients may promote a sustained inflammatory response. Reinhold et al.^5^ suggested that fibrinogen’s interaction with macrophages and microglia could lead to chronic neuroinflammation, synaptic dysfunction, and cognitive impairment.

Conversely, proteins related to antioxidant defense mechanisms were significantly downregulated in the COVID-19 group, particularly extracellular superoxide dismutase (SOD3). This suggests an increased oxidative stress environment, a key factor in COVID-19 pathophysiology.^20^ The reduction of SOD3 is concerning, as it plays a crucial role in mitigating oxidative damage by neutralizing superoxide radicals. The imbalance between oxidative stress and antioxidant defense could contribute to neuronal injury and neuroinflammation, potentially leading to long-term neurological sequelae, as observed in post-COVID syndrome.^21^ Furthermore, Zinc Alpha-2 Glycoprotein (AZGP1), a protein with anti-inflammatory properties, was upregulated in our study. AZGP1 has been implicated in metabolic regulation and immune modulation, particularly through interactions with Transforming Growth Factor Beta (TGF-β), a key regulator of neuroinflammation.^6,17^ The role of AZGP1 in COVID-19 remains poorly understood, but its increased expression may reflect a compensatory mechanism to counteract excessive inflammation.

The differential expression patterns observed in our study highlight the multifaceted impact of SARS-CoV-2 on the CNS. The interplay between neuroinflammation, coagulation dysregulation, and oxidative stress suggests potential mechanisms underlying the neurological complications associated with COVID-19, including cognitive impairment, headache, and neurodegenerative processes.^2,4,6,11,15,17,22–29^ Our findings align with previous studies reporting persistent neurological symptoms in post-COVID patients,^31^ raising concerns about prolonged CNS involvement. The observed proteomic alterations in CSF may serve as potential biomarkers for identifying patients at risk for long-term neurological sequelae. However, further studies with larger cohorts and longitudinal analyses are needed to validate these findings and explore their clinical implications.

By integrating proteomic profiling with the existing literature, our study highlights both well-established mechanisms and novel protein candidates that may be relevant to the pathophysiology of COVID-19-related neurological complications. While some of the identified alterations align with previously reported data, others provide new insights into potential biomarkers and therapeutic targets. Further investigations, particularly longitudinal studies and functional assays, will be essential to elucidate the precise role of these proteins in disease progression and long-term neurological outcomes.

## Conclusions

This study contributes to the emerging understanding of COVID-19 as a systemic disease with significant neuroinvasive potential. The distinct proteomic signature in CSF from patients highlights the need for a comprehensive approach to COVID-19 treatment that addresses not only the respiratory but also neurological consequences of the disease. Future studies should validate these findings in a larger cohort and explore the functional implications of these differentially expressed proteins involved in the neuroinflammatory response and neurological pathology related to COVID-19. Furthermore, longitudinal studies assessing protein levels at different stages of the disease may provide insights into the progression of CNS involvement and the recovery process.

This study contributes to the emerging understanding of COVID-19 as a systemic disease with significant neuroinflammatory potential, even in the absence of direct viral detection in the central nervous system. The distinct proteomic signature identified in CSF samples from rigorously selected patients highlights the need for a comprehensive approach to COVID-19 treatment that addresses not only the respiratory but also the neurological consequences of the disease.

While the exploratory nature of the study and the relatively small sample size limit generalizability, they do not undermine the internal consistency and biological plausibility of the findings. Rather, these results should be viewed as a starting point for hypothesis generation and future validation. Subsequent studies with larger, nonpooled cohorts and integrated virological assessments are essential to confirm these observations and assess their clinical relevance across different stages of COVID-19. Additionally, longitudinal analyses may help distinguish between transient inflammatory responses and long-term neurological sequelae.

By uncovering proteomic shifts in the CSF in the absence of confirmed viral RNA, this study underscores the importance of host-driven mechanisms in neurological manifestations of the CSF-induced cellular changes in COVID-19.

## Limitations

A key limitation of this study is the absence of viral RNA detection in CSF samples, which could have provided direct evidence regarding the presence or translocation of SARS-CoV-2 across the blood-brain or blood-CSF barriers. While proteomic and immunological alterations in the CSF strongly suggest barrier dysfunction and neuroinflammation, the detection of viral RNA, though inconsistently reported in the literature, would have added an additional layer of confirmation to the proposed mechanisms. The lack of RNA testing in this context restricts the ability to definitively rule out or in the possibility of direct viral invasion, even if such events appear to be rare and not necessarily required to trigger neurological manifestations. Therefore, future studies incorporating simultaneous proteomic and virological analyses may help clarify the interplay between the viral presence and immune-mediated CNS responses in COVID-19.

## Material and Methods

### Obtaining Clinical Samples

Cerebrospinal fluid (CSF) was collected via lumbar puncture from 11 adult patients (male and female) in a supine position, all of whom were in severe condition and hospitalized with confirmed COVID-19 at a private hospital in Recife, Pernambuco, in the northeastern region of Brazil. The control group consisted of 5 adult patients with negative COVID-19 tests, also supine, who underwent the lumbar puncture procedure for diagnostic purposes unrelated to the study. Sample pools were prepared by aliquoting 150 μL from each sample into 2 mL LoBind tubes (Eppendorf, ref-0030108450). The local research ethics committee approved the study (approval number 5,353,297), and all patients provided written informed consent before undergoing lumbar puncture. This study adhered to the STROBE guidelines for the reporting of observational studies.

The COVID-19 positive patients were hospitalized and undergoing treatment. The patients’ clinical parameters are detailed in Table S2.

### Sample Preparation

Initially, the CSF samples were subjected to quantification of total proteins using the Pierce BCA protein assay kit method (Thermo Scientific, ref: 23225), where a quantification of 100 μg of proteins was established in each sample in a maximum volume of 100 μL. After quantification, the samples were subjected to protein precipitation and removal of nonprotein interferents, using the clean up kit (Cytiva ref: 80–6484–51). Then, the protein pellets obtained in the previous step went to the proteolytic digestion step.

During digestion, pellets from each sample were added and resuspended with 50 μL of 8 M Urea to break hydrophobic interactions. Then, 2.5 μL of 100 mM dithiothreitol DTT (Cytiva, ref: 17–1318–02) was added and incubated for 30 min at 30 °C to reduce disulfide bonds. Immediately afterward, 2.5 μL of 300 mM iodoacetamide (Cytiva, ref: 25900066) were added and incubated for 30 min at room temperature away from light to promote the alkylation of previously reduced disulfide bonds.

Continuing the procedure, 350 μL of 50 mM ammonium bicarbonate (Sigma-Aldrich), pH 7.8, was added. Then, 10 μL of trypsin gold Promega (0.5 μg/μL) was aliquoted and incubated in a water bath at 37 °C overnight for 18 h. The tubes were centrifuged at 11,000 × *g* for 10 min at 4 °C. The supernatants were collected in LoBind tubes (Eppendorf ref-0030108450), followed by volume reduction in the SpeedVac and stored in the ultrafreezer at −80 °C until use.

### LC-MS/MS Analysis

For high-definition LC-MS/MS analyses, a high-performance liquid chromatography system was used using the M-Class ACQUITY UPLC nanoflow system (Waters Corporation, USA), which was coupled to a mass spectrometer. Hybrid quadrupole time-of-flight (ESI-qTOF MS/MS) model SYNAPT XS (Walters). For the fractionation of previously digested tryptic peptides, we used three columns as our stationary phases, the first dimension being a 1D column (5 μm nanoEase M/Z Peptide BEH130 C18, 300 μm × 50 mm) operating at 2 μL/min.

Two mobile phases were used: mobile phase A (water and 0.1% (v/v) formic acid) and mobile phase B (acetonitrile). In this dimension, peptides were eluted in 5 fractions of mobile phase B (11.4, 14.7, 17.4, 20.7, and 50%) over a period of 70 and 9.5 min charging rate.

Subsequently, in a second dimension, a Trap column (5 μm NanoEase M/Z Symmetry C18, 180 μm × 20 mm) coupled to an analytical column (1.8 μm nanoEase M/Z HSS C18 T3, 75 μm × 150 mm) was operated at 0.4 μL/min and a temperature of 35 °C. Then, the peptides were eluted in two flows, first with a rate of 3 to 45% mobile phase B for 46 min and immediately afterward with 90% mobile phase B lasting 4 min. Then, a flow equilibration was performed with 3% mobile phase B for 20 min.

For MS/MS analyses in the mass spectrometer, spectra with the mass/charge ratio (*m*/*z*) were acquired in positive operating mode and resolution of 30,000, fwhm. The ionization source adapted for nanoflow (ESI Low Flow) was operated with a capillary voltage of 3 kV, a sampling cone of 40 V, a temperature of 100 °C, and a gas cone of 50 L/min.

The ionic fragmentation step was carried out in a collision chamber (Argon gas) and ionic mobility (using Helium gas) to separate possible isoforms. The time-of-flight (ToF) mass analyzer was calibrated with NaCsI from *m*/*z* 50 to 2000 externally, and a reference signal for the mass blocker GluFibrinopeptide B (*m*/*z* 785.8426) was obtained every 30 s. Analyses were performed in triplicate with the UDMSE data acquisition mode.

### Proteomic Data Analysis

The raw mass spectra obtained from LC-MS/MS were processed and deconvoluted using PROGENESIS QI software (Nonlinear Dynamics), Waters, version 4.7. For the database, we used UniProt (Universal Protein Knowledgebase) with the proteome of the species Homo sapiens (downloaded on October 28, 2023). The processing parameters included trypsin cleavage specificity, fixed carbamidomethyl modification for cysteine (Cys), variable modification for methionine oxidation (Met), and a maximum protein mass value of 650 kDa.

The search criteria were set with the following thresholds: a minimum of 2 peptides per protein, a minimum of 2 fragments per peptide, a minimum of 5 fragments per protein, and a false discovery rate (FDR) of 1.0 for protein identification.

We applied the Hi-label-free method for relative quantification, selecting the three most abundant peptides for each identified protein. Only proteins with confidence interval values and frequency scores above 99% were considered acceptable for a database–based investigation.

### Functional Analysis

Functional annotation analyses were performed in the Python environment with the OmicScope package (v.1.2.2), using the Progenesis method and default parameters for enrichment obtained from the Enrich library.^30^ The imported functional annotation libraries were obtained from Gene Ontology (GO) data and used for analyses of biological processes, cellular components and molecular functions. To analyze the biological action pathways, information imported from the REACTOME^18^ database was used.

Protein–protein interaction analysis was performed using Cytoscape version 3.10.2. Interaction maps were created using the UniProt accession code—accession identifier for differentially expressed proteins (DEPs) using the STRING database (http://string-db.org, version 11.5). Using the Omics visualizer plugin, it was possible to show which up- and downregulated proteins interacted in the network through the log2 (fold change) values of each protein. We used as parameters the Homo sapiens filter as the species in question and a cutoff confidence score of 0.70. The MCODE plugin was used to obtain the most interacting protein clusters in the main protein network, with a statistical confidence score of 0.60.^32^

### Statistical Analysis

The OmicScope package (v.1.2.2) was the tool used for statistical analysis of the data imported by the Progenesis method of raw data analysis.^30^ The analyses were performed in a Python environment, and the following parameters were used: minimum fold charge value = 1.5 and false discovery rate (FDR) = 0.01. Proteins with statistical significance were considered those whose adjusted *p*-value = <0.05, and to define differentially expressed proteins (DEPs), a fold change value of 1.5 on the logarithmic scale at base 2 (log2 fold change) was used.

After this step, it was possible to confirm which proteins were differentially expressed (DEP’s) and draw a volcano plot, multiscatter plot with Pearson correlation values, and principal component analysis (PCA) graphs. To build the heat map, the rows containing the data were filtered based on a categorical column, and then the Z-score values were determined.

In addition, we calculated the effect size (Cohen’s *d*) for a subset of representative DEPs using normalized abundance values from the COVID-19 and control groups to evaluate the strength of group-level differences in protein abundance.

## Supplementary Material



## Notes

(Table S1) List of differentially expressed proteins (DEPs) identified by proteomic analysis, including UniProt accession numbers, adjusted *p*-values, log2 fold change values, and statistical parameters (FDR 0.01, *p* ≤ 0.05); (Table S2) clinical parameters of COVID-19 patients, including age, sex, initial lumbar puncture pressure, cell count, protein and glucose levels in CSF, and immunological test results (PDF)
