# Supplementary material for: Cerebrospinal Fluid Proteomic Profiling Reveals Proteins Associated with Neuroinflammatory Response in COVID-19 Patients
Source: ACS Omega. 2025 Jun 11;10(24):25489–97. doi: 10.1021/acsomega.5c00707 (PMC12199065; doi:10.1021/acsomega.5c00707)
Supplement: Supplementary file 1 [file ao5c00707_si_002.pdf]

Cerebrospinal fluid proteomic profiling reveals proteins associated with neuroinflammatory response in COVID-19 patients

Juliana Ramos de Andrade[1], Josivan Barbosa de Farias[1]*, Maria Luiza de Lima Vitorino[1], Fernando Tenório Travassos[2], Roberto Afonso da Silva[1], José Luiz de Lima Filho[1], Marcelo Moraes Valença[1]

[1]Keizo Asami Institute, Federal University of Pernambuco. Av. Prof. Moraes Rego, 50670-901, Recife, Pernambuco, Brazil.

[2]Fernando Travassos Laboratory, Empresarial Thomas Edison - Av. Gov. Agamenon Magalhães, 4775 - 50070-160, Boa Vista, Recife, Pernambuco, Brazil.

***Corresponding Author**:

Josivan Barbosa de Farias, Keizo Asami Institut, Federal University of Pernambuco, Av. Prof. Moraes Rego, 50670-901. Recife, Pernambuco, Brazil; Email: josivan.bfarias@ufpe.br

Table S1: List of differentially expressed proteins (PDEs). In the statistical analyses, the following parameters were considered: log2 fold change = log 2 (1.5), p value ≤ 0.05 and False Discovery Rate (FDR) = 0.01.

Upregulated proteins				
Gene name	***Accession (Uniprot)***	***P value Adjusted***	***-log10 (p Adjusted)***	***log2(Fold change)***
LBP	*P18428*	*0,001153654*	*2,937924488*	*5,710939444*
EXD3	*Q8N9H8*	*0,00035112*	*3,454544923*	*3,50885685*
DNAH17	*Q9UFH2*	*0,000332172*	*3,478636477*	*2,990846051*
FGB	*P02675*	*0,000939114*	*3,027281509*	*2,753869091*
LDHA	*P00338*	*0,000595738*	*3,224945061*	*2,33147028*
APOC3	*P02656*	*0,002661279*	*2,574909633*	*2,265214095*
AMBP	*P02760*	*0,000878902*	*3,056059737*	*2,218081189*
C1R	*P00736*	*0,000888648*	*3,051270134*	*2,195230637*
AZGP1	*P25311*	*0,041460189*	*1,382368722*	*1,937430333*
TRDV2	*A0JD36*	*0,00035112*	*3,454544923*	*1,881735355*
FGA	*P02671*	*0,000393838*	*3,404682373*	*1,823025063*

**-**	*P0DOX7*	*0,000969016*	*3,013668957*	*1,750974279*
**IGKV3-20**	*P01619*	*0,014872472*	*1,827616849*	*1,72902518*
**CFD**	*P00746*	*0,000846498*	*3,072373865*	*1,711416535*
**C4B_2**	*P0C0L5*	*0,000969016*	*3,013668957*	*1,709180873*
**C4A**	*P0C0L4*	*0,000969016*	*3,013668957*	*1,709180873*
**PKM**	*P14618*	*0,001022433*	*2,99036501*	*1,698990638*
**C9**	*P02748*	*0,022911229*	*1,639951622*	*1,639087696*
**IGHV5-51**	*A0A0C4DH38*	*0,005179557*	*2,285707403*	*1,569015127*
**SERPINA3**	*P01011*	*0,000507846*	*3,29426758*	*1,484830869*
**CD14**	*P08571*	*0,048625555*	*1,313135432*	*1,451314908*
**HP**	*P00738*	*0,002509434*	*2,600424282*	*1,299532876*
**SERPINA1**	*P01009*	*0,013040274*	*1,884713288*	*1,237850764*
**CALR**	*P27797*	*0,013181307*	*1,880041532*	*1,229619981*
**IGHV3-72**	*A0A0B4J1Y9*	*0,000422504*	*3,374169439*	*1,155390343*
**IGHG3**	*P01860*	*0,001120128*	*2,95073232*	*1,081194229*
**CFI**	*P05156*	*0,025898299*	*1,586728759*	*1,058263004*
**TIMP1**	*P01033*	*0,002509434*	*2,600424282*	*1,043612554*
**CEP192**	*Q8TEP8*	*0,001926913*	*2,715137885*	*0,963280768*
**ORM1**	*P02763*	*0,029216124*	*1,534377407*	*0,958399477*
**APOD**	*P05090*	*0,008731568*	*2,058907774*	*0,958208764*
**BLVRA**	*P53004*	*0,002547441*	*2,593895793*	*0,955912091*
**F2**	*P00734*	*0,034423762*	*1,46314167*	*0,945443083*
**IGLV3-1**	*P01715*	*0,00035112*	*3,454544923*	*0,943149135*
**IGFBP6**	*P24592*	*0,00035843*	*3,445595187*	*0,936963198*
**IGKC**	*P01834*	*0,016413498*	*1,784798848*	*0,906697091*
**CP**	*P00450*	*0,049434941*	*1,305965981*	*0,881327004*
**CHI3L1**	*P36222*	*0,010661927*	*1,972164305*	*0,834089571*
**PGLYRP2**	*Q96PD5*	*0,02251955*	*1,64744029*	*0,788167616*
**SPANXN4**	*Q5MJ08*	*0,002661279*	*2,574909633*	*0,698446329*

APOA1	*P02647*	*0,002509434*	*2,600424282*	*0,637722969*
C7	*P10643*	*0,042307857*	*1,373578969*	*0,629154491*
C1QB	*P02746*	*0,020387208*	*1,690642236*	*0,620812315*
EFEMP1	*Q12805*	*0,049434941*	*1,305965981*	*0,609333502*
GOLM1	*Q8NBJ4*	*0,002509434*	*2,600424282*	*0,586235884*

Dowregulated proteins

Gene name	***Accession (Uniprot)***	***P value Adjusted***	***-log10 (p Adjusted)***	***log2(Fold change)***
SERPINF2	*P08697*	*0,015006456*	*1,823721847*	*-0,587066936*
SERPINF1	*P36955*	*0,001872511*	*2,72757551*	*-0,613697851*
NCAM1	*P13591*	*0,001293158*	*2,888348451*	*-0,654422111*
ALB	*P02768*	*0,044614054*	*1,350528309*	*-0,661748813*
CST3	*P01034*	*0,001872511*	*2,72757551*	*-0,710635516*
GOLGA8IP	*A6NC78*	*0,000393838*	*3,404682373*	*-0,773275731*
GC	*P02774*	*0,003514024*	*2,454195266*	*-0,802787252*
CDH13	*P55290*	*0,00035112*	*3,454544923*	*-0,865661986*
FAM110D	*Q8TAY7*	*0,027585442*	*1,559320055*	*-0,946874891*
SCG5	*P05408*	*0,000939114*	*3,027281509*	*-0,952311664*
APLP1	*P51693*	*0,042307857*	*1,373578969*	*-0,980717753*
TF	*P02787*	*0,014872472*	*1,827616849*	*-0,983325565*
KMT2B	*Q9UMN6*	*0,002509434*	*2,600424282*	*-1,044702403*
TTR	*P02766*	*0,015006456*	*1,823721847*	*-1,090026608*
FAHD1	*Q6P587*	*0,000764572*	*3,116581562*	*-1,099871562*
PENK	*P01210*	*0,00035112*	*3,454544923*	*-1,119520133*
LRG1	*P02750*	*0,004939755*	*2,306294627*	*-1,133414792*
SSH1	*Q8WYL5*	*0,020640136*	*1,685287448*	*-1,203808897*
HBB	*P68871*	*0,008731568*	*2,058907774*	*-1,251903303*
NRCAM	*Q92823*	*0,028869539*	*1,539560154*	*-1,259729552*
PTGDS	*P41222*	*0,016890372*	*1,772360783*	*-1,308431069*

OPCML	*Q14982*	*0,00035112*	*3,454544923*	*-1,330245209*
VTN	*P04004*	*0,022911229*	*1,639951622*	*-1,33410127*
SEMA7A	*O75326*	*0,000969016*	*3,013668957*	*-1,458234196*
VGF	*O15240*	*0,000837697*	*3,076912822*	*-1,592195569*
ECM1	*Q16610*	*0,003312891*	*2,479792913*	*-1,647388335*
SOD3	*P08294*	*0,026299601*	*1,580050837*	*-1,718060954*
HBA2	*P69905*	*0,010592259*	*1,975011415*	*-1,723475168*
CUTA	*O60888*	*0,041460189*	*1,382368722*	*-2,52260461*
IGFBP7	*Q16270*	*0,00035112*	*3,454544923*	*-2,66668602*
FOXI3	*A8MTJ6*	*0,004939755*	*2,306294627*	*-3,626890334*

Table S2. Clinical parameters of COVID-19 patients. The table includes demographic data (age, sex), initial lumbar puncture pressure, total cell count, differential cell count, protein and glucose levels in the cerebrospinal fluid (CSF), and immunological test results for each patient.

Pacient	Age	Sex	Condition	Initial pressure	Total cell count	Differential cell count	Protein	Glucose	Immunological test
**1**		M	Alert / tense	18	7	90L 10M	72	54	VDRL - nr
**2**	26	M	Coma	22	57	20L 10M 70N	517	148	VDRL - nr
**3**	26	M	Coma	25	1	90L 10M	50	136	CMV/ HSV: nr / ADA: 3.0 / CI: n / ZIEL: n / GRAM: a
**4**	30	F	Tense	18	3	90L 10M	40	42	CMV / HSV / EBV / HIV / HZ: nr / CI: n / CLT: nr
**5**	32	F	Tense	19	1	90L 10M	46	56	CMV / HSV / EBV / HIV / HZ : nr / ZIEL / CI: nr / ADA: 2.2
**6**	20	M	Coma	25	1	90L 10M	163	85	CI: n
**7**	21	F	Alert / calm	10	13	90L 10M	148	59	VDRL: nr
**8**	40	M	Alert	25	3	100% LM	31	53	HSV / VDRL: n / HZ: p
**9**	35	M	Coma	15	1	100% LM	26	137	EFP: 5,47,5,6,17,4,16 A / G: 0,98 / EFP SORO 42,5,14,18,21 A/G 0,72

**10**	49	M	Alert	19	1	100% LM	46	53	HSV / CMV / HIV: nr / EFP 6,57,4,6,16,-,11 A/G 1,54 / EFP SORO 43,5,8,16,28 A/G 0,75 / I IGG 0.29
**11**	54	M	Alert	30	1	100% LM	69	44	VDRL / CI / HSV / CMV: nr / INDIFE DE IGG 0,65

nr – non-reactive, n – negative, p – positive, a – absent, VDRL - Venereal Disease Research Laboratory, CMV – Cytomegalovirus, HSV - Herpes simplex virus, ADA - Adenosine deaminase, CI - China ink, EBV - Epstein-barr virus, HZ – Herpes zoster, HIV - Human immunodeficiency virus, CLT - Crypto-la-test
